# Phenotypic Modulation of Macrophages and Vascular Smooth Muscle Cells in Atherosclerosis—Nitro-Redox Interconnections

**DOI:** 10.3390/antiox10040516

**Published:** 2021-03-26

**Authors:** Justine Bonetti, Alessandro Corti, Lucie Lerouge, Alfonso Pompella, Caroline Gaucher

**Affiliations:** 1 CITHEFOR, Université de Lorraine, F-54000 Nancy, France; justine.bonetti@univ-lorraine.fr (J.B.); lucie.lerouge9@etu.univ-lorraine.fr (L.L.); caroline.gaucher@univ-lorraine.fr (C.G.); 2 Department of Translational Research NTMS, University of Pisa Medical School, 56126 Pisa, Italy; alessandro.corti@med.unipi.it

**Keywords:** atherosclerosis, macrophages, vascular smooth muscle cells, differentiation/de-differentiation, signalling pathways, nitric oxide

## Abstract

Monocytes/macrophages and vascular smooth muscle cells (vSMCs) are the main cell types implicated in atherosclerosis development, and unlike other mature cell types, both retain a remarkable plasticity. In mature vessels, differentiated vSMCs control the vascular tone and the blood pressure. In response to vascular injury and modifications of the local environment (inflammation, oxidative stress), vSMCs switch from a contractile to a secretory phenotype and also display macrophagic markers expression and a macrophagic behaviour. Endothelial dysfunction promotes adhesion to the endothelium of monocytes, which infiltrate the sub-endothelium and differentiate into macrophages. The latter become polarised into M1 (pro-inflammatory), M2 (anti-inflammatory) or Mox macrophages (oxidative stress phenotype). Both monocyte-derived macrophages and macrophage-like vSMCs are able to internalise and accumulate oxLDL, leading to formation of “foam cells” within atherosclerotic plaques. Variations in the levels of nitric oxide (NO) can affect several of the molecular pathways implicated in the described phenomena. Elucidation of the underlying mechanisms could help to identify novel specific therapeutic targets, but to date much remains to be explored. The present article is an overview of the different factors and signalling pathways implicated in plaque formation and of the effects of NO on the molecular steps of the phenotypic switch of macrophages and vSMCs.

## 1. Introduction

**Figure 1:**
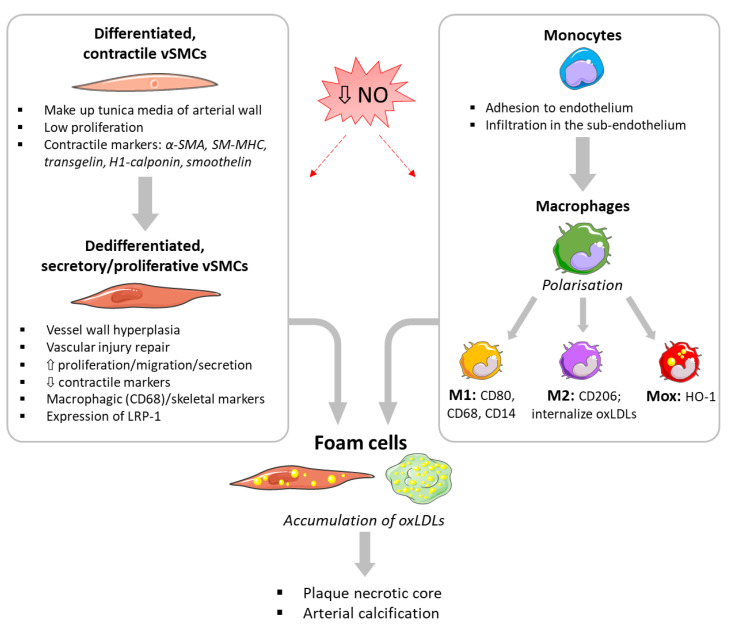
Summary of the mains steps in formation of foam cells in atherosclerotic plaques. vSMCs: vascular smooth muscle cells; α-SMA: alpha-smooth muscle actin; SM-MHC: smooth muscle-myosin heavy chain; LRP-1: pro-low density lipoprotein receptor-related protein 1; M1: M1 macrophages; M2: M2 macrophages; oxLDLs: oxidized low-density proteins; Mox: Mox macrophages; HO-1: heme oxygenase 1.

Cardiovascular diseases are the major cause of death worldwide, being responsible for 17.9 million deaths (31% of the global death rate) in 2016 [1]. From these deaths, 85% were caused by heart attacks and strokes, which can be the consequence of atherosclerosis progression and plaque rupture. Atherosclerosis—the leading coronary arteries disease—is a chronic, silent and slowly developing pathology, mainly affecting arteries of medium–large diameter and characterised by the formation of atheroma plaques as a result of excess plasmatic low-density lipoprotein (LDL) concentrations. The accumulation of LDLs in the blood stream results in their infiltration and oxidation in the sub-endothelium, leading to vascular wall inflammation and oxidative stress. This deleterious environment induces endothelial dysfunction, in turn leading to decreased bioavailability of nitric oxide (NO) due to both decreased synthesis and increased catabolism. NO is a gaseous, lipophilic, free radical mediator with a short half-life (<5 s), synthesised by endothelial NO synthases (eNOS) and involved in the maintenance of vascular homeostasis. Endothelial dysfunction and decreased NO bioavailability induce adhesion/infiltration in the sub-endothelium of circulating monocytes, as well as migration/proliferation of vascular smooth muscle cells (vSMCs) from the arterial tunica media (Figure 1). Oxidised low-density lipoproteins (oxLDLs) are then phagocytised by macrophages or internalised by vSMCs, thus originating the typical “foam cells” participating in the development of the plaque necrotic core (Figure 1).

NO displays antiaggregant and vasorelaxant properties, concurring to maintain the anti-inflammatory phenotype of endothelial cells as well as the non-proliferative and non-migratory phenotype of vSMCs. The classical NO mechanism of action is mediated by the nitrosylation (coordination of NO to a metal) of haemoproteins, such as soluble guanylyl cyclase (sGC) producing cyclic guanosine monophosphate (cGMP), which is mostly responsible for the antiaggregant and vasorelaxant properties of NO. However, NO signalling is also mediated through protein *S*-nitrosation, a post-translational modification of high or low molecular weight proteins or peptides involving the formation of a covalent bond between NO and a reduced thiol of a cysteine residue. *S*-Nitrosated proteins and peptides are also storage and transport forms of NO: indeed, despite its short half-life, in this way NO is able to act at a distance from its sites of synthesis. The *S*-nitrosation process extends the NO half-life from 45 min up to several hours and limits the oxidative/nitrosative stress caused by NO oxidation into peroxynitrite ions (ONOO^−^). In vivo, *S*-nitrosothiols, such as *S*-nitrosoalbumin, *S*-nitrosohemoglobin and *S*-nitrosoglutathione (GSNO), are the main physiological forms of NO storage and transport. Finally, NO action can also be mediated through protein or lipid nitration, a marker of nitrosative stress characterized by the addition of a nitro group to a tyrosine residue or a fatty acid chain.

NO has anti-inflammatory and anti-proliferative actions, and its depletion during atherosclerosis will favour monocyte adhesion and infiltration in the sub-endothelium, as well as vSMCs migration/proliferation and their switch from a contractile to a secreting phenotype. Monocytes infiltrating the sub-endothelium are exposed to oxLDLs, and differentiate into macrophages of different subtypes depending on the prevalent microenvironment. At first monocytes differentiate into naïve M0 macrophages, which can then become polarised into M1 (pro-inflammatory), M2 (anti-inflammatory) or Mox macrophages (oxidative stress phenotype). Indeed, macrophages show a high plasticity, and a dynamic switch between the mentioned phenotypes. M1 and M2 macrophages express specific phenotypic markers, e.g., LDL surface receptors CD80, CD68 and CD14 for the M1 phenotype [2]; the CD206 mannose receptor for the M2 phenotype [3]; or heme oxygenase 1 (HO-1) for Mox phenotype [4] (Figure 1). The balance of the M1/M2 population is modified along the atherosclerotic plaque development, varying from a M2 prevalence in stable plaques to a M1 prevalence in vulnerable ones [5,6,7]. Phagocytosis of oxLDLs by macrophages leads to the formation of foam cells, composing the lipidic necrotic core of plaques (Figure 1). M2 macrophages internalise more lipids than M1 macrophages, due to a lower expression of cholesterol efflux proteins [7]. The higher oxLDLs loading of M2 causes eventually a shift towards an M1 phenotype [8,9]. However, other studies found an increased oxLDLs uptake in M1-like macrophages, even when cholesterol efflux proteins are upregulated [10]. Mox macrophages, a distinct phenotype from M1 and M2 macrophages, derived from M0 macrophages, phagocyted oxLDLs and protect from oxidative stress. Treatment of M1 or M2 macrophages with oxLDLs causes also a further switch to the a specific Mox phenotype [11].

In healthy arteries, vSMCs are located in the tunica media of the vessel wall where they are responsible of vasoactivity (contraction/relaxation), vascular diameter and blood pressure. Differentiated contractile vSMCs have low migration and proliferation abilities. They express specific contractile markers like α-smooth muscle actin (α-SMA), smooth muscle myosin heavy chain (SM-MHC), transgelin, H1-calponin and smoothelin (Figure 1). However, unlike other muscle cells, vSMCs do not terminally differentiate and retain a high level of plasticity while remaining in a quiescent phenotype. In response to vascular injury and inflammatory stimuli, such as during atherosclerosis, contractile vSMCs are able to undergo de-differentiation to a secretory/proliferative (“pro-atherosclerotic”) phenotype, which is implicated in vascular wall hyperplasia occurring during progression of atherosclerotic plaques [12]. Indeed, such de-differentiation has a physiological function in helping vascular repair after injury and is the same mechanism as observed in embryonic angiogenesis, neovascularisation and vascular remodelling [13]. De-differentiated vSMCs are characterised by a decreased expression of contractile markers and increased proliferation, migration and secretory abilities, and can even express markers of macrophagic as well as skeletal muscle phenotypes. De-differentiated vSMCs act in all steps of atherosclerosis development, until the formation of advanced plaques presenting a necrotic core and varying degrees of calcification. The vSMCs’ phenotypic switch allows their migration and infiltration from the media to intima due to secretion of matrix metalloproteinases (MMP-2, MMP-9) [14], as well as their capacity to internalise oxLDLs through the expression of the pro-low density lipoprotein receptor-related protein 1 (LRP-1) (Figure 1). During this process, the nuclear localisation of NF-κB is increased, leading to secretion of pro-inflammatory cytokines and promotion of vSMCs dedifferentiation.

During their phenotypic switch, vSMCs acquire macrophagic properties and express macrophage markers, such as CD68 [15], as well as mesenchymal stem cells markers (e.g., Sca1 and CD105) [16,17], suggesting the presence of a vSMC progenitor population within the vessel wall that proliferates and accumulates along with the development of atherosclerosis (Figure 1). The switch to a macrophage-like phenotype is likely driven by lipids accumulation and is the main step in the formation of foam cells and the necrotic core of plaques [16]. Foam cells encountered in plaques have been shown in fact to derive from both macrophages and vSMCs, through oxLDLs accumulation [18,19,20]. Allahverdian et al. previously demonstrated that, of the foam cells present in human plaques, 40% are of vSMC origin vs. 60% of a macrophagic one [21]. 

Many signalling pathways triggered by inflammatory and/or oxidative stimuli are implicated in the phenotypic switch of vSMCs, as well as in the differentiation of monocytes and the polarisation of macrophages. Activation of these signalling pathways occurs in parallel to a decrease in NO bioavailability. NO is known to promote M1 polarisation toward the M2 phenotype [22,23], and has been held responsible for the maintenance of the non-proliferative/non-migratory phenotype of vSMCs. However, no direct proof was provided for the alleged ability of NO to counteract the vSMCs’ phenotypic switch. The present review is an overview and an appraisal of the current knowledge on the signalling pathways involved in vSMC and monocyte/macrophage differentiation/polarisation, with particular reference to NO-dependent signalling.

## 2. Modulation of Key Determinants of Cell Phenotype by Redox/Inflammatory Signalling

Under physiological conditions, reactive oxygen species (ROS) produced at low concentration are key redox regulators of cell functions in response to extracellular and intracellular stimuli. At first, cell cycle phases as well as assembly of the mitotic spindle were associated with variations in concentration of soluble thiols [24,25]. In particular, glutathione was shown to accumulate during the G_2_ phase and the mitotic phase [26], in which the ROS concentration was shown to be 3-fold higher than in the G_1_ phase [27]. The redox environment as well as inflammatory stimuli appear thus to control the cell cycle, especially transitions between phases [28,29,30,31].

The implications of ROS generating as well as antioxidant systems along the different steps of atherosclerosis progression (endothelial dysfunction, LDLs oxidation and accumulation, plaque formation) have been recently reviewed [32]. Paracrine signalling by growth factors, cytokines and hormones, such as platelet-derived growth factor (PDGF) [33,34], epithelial growth factor (EGF), transforming growth factor β (TGF-β), tumour necrosis factor-α (TNF-α) [35] and angiotensin II [36], activates specific cell membrane receptors and induce ROS production. ROS can then reversibly oxidise thiol groups on regulatory proteins to disulfides, mixed disulfides or sulfoxides, as well as zinc-fingers to disulfides, or methionine residues to methionine [37].

### 2.1. Platelet-Derived Growth Factor (PDGF)

PDGF is one of the most robust phenotype-modulating agents, and a primary regulator of vSMCs proliferation in response to a multitude of stimuli like hypoxia, thrombin, growth factors (including PDGF itself) or cytokines. PDGF is produced as a dimer of four polypeptide chains, linked by disulphide bonds and encoded by four different genes. Five possible dimeric forms of PDGF have been identified: PDGF-AA, PDGF-AB, PDGF-BB, PDGF-CC and PDGF-DD. PDGF-A and PDGF-B monomers undergo activation (N-terminal pro-domain removed) during their intracellular transport for secretion [38]. PDGF-C and PDGF-D monomers are secreted as latent factors, requiring an activation by extracellular proteases [39,40].

Among the PDGF isoforms, PDGF-BB dimer is known to drive the Raf/Ras/MEK/ERK signalling pathway. Vascular injury and chronic arterial diseases result in PDGF-BB production as a signal for vSMCs de-differentiation, helping the remodelling of blood vessels. Indeed, PDGF-BB decreases the expression of contractile phenotype markers (calponin and α-smooth muscle actin) and upregulates the expression of synthetic markers osteopontin and vimentin. Furthermore, PDGF-BB prevents oxidative stress-induced protein damage and cell death by promoting vSMC autophagy, essential for cell survival [41]. PDGF-BB is central for vSMC proliferation and migration regulation linked with ROS signalling and JAK/STAT signalling. NADPH oxidase (NOX), producing superoxide anions, in fact participates in signalling cascades regulating PDGF-BB-induced proliferation, while JAK inhibition abolishes PDGF-stimulated vSMCs growth [42].

PDGF-BB is also implicated in the modification of the extracellular matrix surrounding vSMCs. Contractile vSMCs are surrounded by type I and IV collagens and proteoglycans. However, along atherosclerosis development, vSMCs secrete type I, VI and VIII collagens, as well as MMPs promoting their migration, followed by upregulation of synthesis of interstitial matrix components such as elastin, fibronectin, osteopontin or tenascin. Collagen deposits account for 60% of the total protein in atherosclerotic plaques, and drive vSMC migration [43]. PDGF-BB promotes the synthesis of type VI collagen α1 chain, increasing vSMCs viability and migration capacity, as well as fibronectin and MMPs through the activation of Akt/mTOR signalling [44].

Addition of PDGF-BB to vSMCs suppresses SM-MHC mRNA/protein expression, but treatment with NO donors (FK409 or *S*-nitroso-*N*-acetylpenicillamine (SNAP)) can reverse this effect [45]. A study with short- (SNAP) and long-term (DETA/NO) NO donors showed that both compounds could inhibit PDGF-induced vSMCs migration in a dose-dependent manner, via blockade of the RhoA pathway [46,47]. De Oliveira et al. also showed that cyclamNO, a NO donor, can inhibit PDGF-BB-induced cell proliferation and migration [48]. CyclamNO prevents vSMCs’ phenotypic switch by reducing (≈60%) the expression of α-SMA induced by PDGF-BB. After a long exposure to SNAP, NO contributed to decrease vSMC proliferation by the attenuation of PDGF-BB-induced PKB-α activation [49]. Moreover, the loss of eNOS activity (eNOS^−/−^ mice) is associated with the activation of the PDGF signalling pathway and with the induction of survivin, an apoptosis inhibitor. eNOS negatively regulates the PDGF-survivin axis, so that proportional flow-dependent luminal remodelling and vascular quiescence are maintained [50].

### 2.2. Transforming Growth Factor-Β (TGF-Β) Superfamily

#### 2.2.1. TGF-β

TGF-β is important in the maintenance of vascular homeostasis and integrity and is produced by all cell types in the arterial wall following a vascular lesion [51]. TGF-β was initially recognised as a de-activating factor for macrophages, with the ability to suppress iNOS expression and to inhibit the production of pro-inflammatory mediators [52], resulting in the suppression of differentiation of monocytes to macrophages [53]. Three isoforms are comprised in the TGF-β family, TGF-β1, -β2 and -β3, secreted as precursors whose biological function is activated by enzymatic cleavage. TGF-β1 and TGF-β3 are expressed in vSMCs, macrophages and foam cells of early human vascular lesions [54]. Indeed, TGF-β contributes to the progression of lipid-rich atherosclerotic lesions by stimulating the production of lipoprotein-trapping proteoglycans, inhibiting vSMCs proliferation and activating proteolytic mechanisms in macrophages [54]. TGF-β regulates CD36 or apolipoprotein E (ApoE) genes implicated in oxLDLs uptake and in cholesterol efflux from foam cells [55]. In addition, TGF-β inhibits foam cells formation and controls cholesterol homeostasis in macrophages via Smad-2 and Smad-3 phosphorylation in macrophages.

Regarding inflammation, TGF-β1 exerts a protective role by inhibiting vSMC proliferation and migration by inhibiting signal transducer and activator of transcription 3 (STAT3) and NF-κB pathways, while at the same time inducing expression of contractile genes and actin re-organisation through an increased p38 MAPK activity [56,57,58,59,60]. Such TGF-β1-driven effects are blocked by inhibitors of RhoA kinase and its target PKN (also known as protein kinase C-related kinase 1 or PRK-1). The TGF-β1/RhoA/PKN triad in fact is the key component of an important intracellular signalling pathway promoting vSMC differentiation. The antiproliferative effects of NO on vSMCs are explained by its interactions with RhoA: *S*-nitrosation of the cysteine residues present in RhoA GTP-binding domain decreases its affinity for GTP, thus suppressing RhoA activity [61,62]. RhoA inhibition by *S*-nitrosation modulates the phosphorylation of myosin light chain, suggesting an implication of cGMP-independent effects of NO on vascular tone as well.

#### 2.2.2. Bone Morphogenetic Proteins (BMPs) and Gremlin-1

BMPs are inflammatory mediators produced by the endothelium in response to shear stress, pro-inflammatory cytokines and oxidative stress [63]. They are members of the TGF-β superfamily, and participate in bone formation, haematopoiesis and cell differentiation during embryogenesis.

In 1993, BMP-2 was first implicated in calcification of human atherosclerotic lesions [64]. Nakaoka et al. demonstrated then that BMP-2 can inhibit vSMC proliferation, and suggested the therapeutic application of BMP-2 to prevent vascular proliferative disorders [65]. On the other hand, BMP-2, BMP-4 and BMP-6 have also been shown to increase plaque formation, oxidative stress, inflammation and endothelial dysfunction [66,67], through the activation of their specific BMP type I and type II (serine/threonine kinase) receptors. BMP receptor signalling has been involved in endothelial dysfunction via inhibition of the Akt-eNOS pathway after induction of phosphatase and tensin homolog (PTEN), as well as in vSMC osteogenic differentiation [68].

BMPs modulate the vSMCs’ phenotype via cross-talk with RhoA/MRTFs pathways, which may contribute to the development of pathological features. Indeed, BMPs promote the nuclear localisation and recruitment of myocardin-related transcription factors (MRTF-A and MRTF-B) to the promoter of the smooth muscle α-actin gene [69]. In particular, BMP-4 decreases SM22-α and α-actin gene expression, indicating vSMC de-differentiation [70].

The inhibition of BMP-2, BMP-4 and BMP-7 binding to BMP type II receptors by Gremlin-1, the physiological antagonist of BMP receptors, reduces Smad activation in the cytoplasm and induces VEGFR-2 expression of VEGFR-2. Gremlin-1 also prevents the recruitment and the activation of macrophages in atherosclerotic plaques by inhibiting the inflammatory mediator MIF [71]. Within vSMC de-differentiation, Gremlin-1 appears to act also independently of the BMP pathway, by reducing the expression of SM22-α, α-actin and calponin genes, as well as by efficiently promoting VEGFR-2-dependent angiogenesis [72].

### 2.3. Fibroblast Growth Factors (FGFs)

The FGFs family comprises 22 members divided into 7 subgroups. FGFs act as signal molecules that bind and activate FGF receptors (FGFRs), a family of tyrosine kinase receptors including four members.

In 1996, Hughes documented FGF/FGFR involvement in atherogenesis and atherosclerotic plaque progression [73]. Indeed, FGF is one of the growth factors released by vSMCs, implicated in their proliferation and phenotypic switch by inducing the secretion of chemokines (e.g., CCL2), a mechanism that could explain the infiltration of leukocytes during atherosclerosis development [74]. In vSMCs, FGF activation of FGFR-1 signalling also upregulates via Src/MEK/MAP kinases the expression of osteopontin, which is implicated in the migration of adventitial fibroblasts [75]. The FGF-2/FGFR-1 interaction increases atherosclerotic plaque instability through NF-κB potentiation and the consequent increase in MMP-9 and iNOS expression in vSMCs [76].

Low concentrations of NO were found to inhibit FGF-2-mediated vSMC migration [77]. On the other hand, high levels of NO induce vSMCs death, resulting in the release of FGF-2, in turn stimulating the proliferation of adjacent endothelial cells. Thus, vSMC damages induced by a high NO concentration may be a trigger of neovascularisation in atherosclerotic plaques [78]. The FGF-2 binding affinity for FGFR-1 is increased by H_2_O_2_, and thus oxidative stress can stimulate vSMC proliferation [79]. FGF-21 in particular has been shown to regulate lipid metabolism, foam cell formation, macrophage migration and inflammatory response by repressing NF-κB signalling [80]. FGF-21 could thus exert a cardioprotective action by inhibiting the initial steps in atherosclerosis.

Finally, a crosstalk between FGF and TGF-β has been observed both in vitro and in vivo, likely contributing to the modulation of the vSMCs’ phenotypic switch. In vitro, the inhibition of FGF signalling increases TGF-β activity, promoting vSMCs differentiation and decreasing their proliferation. In vivo, FGF receptor knock-out mice crossed with ApoE^−/−^ mice present a significant inhibition of atherosclerotic plaque growth [81].

### 2.4. Angiotensin II (AngII)

AngII is the main player in the renin–angiotensin system, regulating renal and vascular homeostasis. The main AngII effects are mediated by the angiotensin II type 1 receptor (AT_1_R) expressed on monocytes, endothelial cells, vSMCs and fibroblasts. Preclinical studies demonstrated that the administration of the AT_1_R antagonist telmisartan for 20 weeks to ApoE^−/−^ mice reduces atherosclerosis progression, with decreased lipid deposition and accumulation of macrophages [82]. In parallel, the plaque collagen content was increased, indicating plaque stabilisation and an overall protective effect of telmisartan [82]. It seems, therefore, that the AngII/AT_1_R signalling can promote progression of atherosclerosis.

Interestingly, NO may counteract AT_1_R function. AT_1_R *S*-nitrosation was in fact shown to decrease its affinity for AngII [83], and to inhibit both AngII-dependent and -independent activation of AT_1_R [84]. AngII also plays a role in atherosclerosis progression by enhancing inflammation, endothelial dysfunction and vSMCs proliferation, thus favouring plaque vulnerability and rupture [85,86]. Decreased NO bioavailability and increased ROS levels can explain the pro-atherogenic effects of AngII [87]. Indeed, AngII activates endothelial NOX and p22phox, leading to the production of superoxide/H_2_O_2_, which, in turn, activates H_2_O_2_-sensitive signalling pathways, leading to vSMC proliferation [88,89]. AngII-induced hypertension in rats was associated with increased NOX-derived O_2_^•–^, endothelial dysfunction and upregulation of AngII receptors [90]. All these effects were blocked by the AT_1_R antagonist losartan, and, in particular, NO can suppress NOX-dependent superoxide production through *S*-nitrosation of the p47phox subunit, resulting in suppression of vascular oxidative stress [91]. Similarly, the knockdown of p22phox, a subunit of the NOX complex, essential for its stability and activity, was shown to inhibit AngII-induced vSMC de-differentiation, proliferation and migration through the inhibition of H_2_O_2_ production, Krüppel-like factor 4 (KLF-4) expression as well as phosphorylation of Akt and activation of ERK1/2 signalling [92]. Indeed, KLF-4 is one of the main transcriptional regulators of vSMCs’ phenotypic switch, and is the end effector of the Akt/ERK1/2 signalling pathway (see Section 4.5).

### 2.5. Oxidized Low Density Lipoproteins (oxLDLs)

Increased levels of circulating oxLDLs are known to promote monocytes adhesion to the endothelium. However, their effects are not limited to the luminal surface, as oxLDLs can penetrate the vascular wall and enhance oxidative stress and inflammation [93]. The latter is responsible for monocytes infiltration in the sub-endothelium and their differentiation into macrophages, expressing the scavenger receptors CD36 as well as lectin-like oxLDL receptor-1 (Lox-1) responsible for oxLDLs binding and uptake. Moreover, oxLDLs induce the phenotypic switch of M1 and M2 macrophages towards the Mox phenotype, i.e., macrophages with a decreased phagocytic activity expressing a panel of antioxidant enzymes under the control of the KLF-2 transcription factor [4]. Mox macrophages represent around 30% of all macrophages found in advanced atherosclerotic lesions of LDLR^−/−^ mice [4].

The majority of foam cells found in plaques are derived from macrophages; however, oxLDLs also promote the vSMCs’ phenotypic switch up to a final metaplasia into typical foam cells. Indeed, oxLDLs, such as oxPAPC (oxidized 1-palmitoyl-2- arachidonoyl-sn-glycero-3-phosphocholine), cause a dose-dependent downregulation of mRNA transcription for α-actin and SM-MHC, two vSMCs phenotypic markers [93]. Moreover, POPVC (1-palmytoyl-2-(5-oxovaleroyl)-sn-glycero-3-phosphocholine) induces pro-inflammatory gene expression, such as chemokine ligand-2 (CCL2), MCP3 and the TNF superfamily, and induces the nuclear translocation of the KLF-4 transcription factor implicated in the repression of vSMC differentiation [93]. De-differentiated vSMCs acquire macrophage-like markers, such as CD68, as well as antigen presentation capacity. On the other hand, such de-differentiated vSMCs cannot perform phagocytosis (only internalisation, lacking digestion steps) and maintain low expression levels of SM-specific markers, such as SM-α-actin [12].

With respect to NO homeostasis, oxLDLs produce a modification of the eNOS/iNOS balance with downregulation of eNOS and sur-expression of iNOS, leading to sur-activation of Lox-1 receptor and NF-kB. The latter originates high concentrations of NO (>1 µM) capable of promoting atherosclerosis progression by increasing LDL oxidation and inflammation, decreasing protective autophagic responses and accelerating the apoptosis of endothelial cells [94]. iNOS inhibition results in the decreased of NO and peroxynitrite ions concentrations after oxLDL treatment, while generation of superoxide is increased, reversing the oxLDL-induced migratory arrest of foam cells [95]. Moreover, high NO concentrations (100 µM) were shown to decrease cholesterol efflux from macrophages through the downregulation of ATP-binding cassette transporter A-1 (ABCA-1), thus increasing the oxLDLs and cholesterol accumulation [96]. The concentration and the type of NO donor used in these experiments is of importance: indeed, SNAP—a *S*-nitrosothiol NO donor—is unable to oxidize LDLs, whereas NO donors sodium nitroprusside and 3-morpholinosydnonimine (SIN-1) induce a strong LDLs oxidation [97]

Overall, the restoration of NO bioavailability at physiological concentrations might be protective against atherosclerosis progression, and pharmacological treatments are obtaining the first results in this perspective. Indeed, treatment of LDLR^−/−^ mice with 0.51 µmol/kg/day with *S*-nitroso-*N*-acetylcysteine allowed a decreased extent of atherosclerotic plaques, of oxidative stress as well as of free circulating cholesterol [98]. Furthermore, NCX6560, a NO-releasing derivative of atorvastatin (a lipid-lowering drug), presented better anti-thrombotic and anti-inflammatory effects than atorvastatin alone [99].

## 3. Signalling Pathways Implicated in Phenotypic Modulation

### 3.1. PI3K/Akt/mTOR Signalling

The phosphatidylinositol-3-kinase (PI3K)/protein kinase B (Akt) signalling pathway, as well as the downstream targets of Akt, play a central role in several cellular processes, such as growth, proliferation, death and differentiation [100,101], and are also implicated in the regulation of vSMC proliferation and contractility. Insulin as well as insulin-like growth factor (IGF) signalling suppress the de-differentiation programme in vSMCs and maintain their contractile phenotype via the classical PI3K/Akt pathway [102]. Active nuclear Akt phosphorylates FOXO-4, a co-repressor protein that interacts with the CarG/SRF/myocardin complex and blocks the expression of differentiation genes FOXO-4 is then exported from the nucleus, allowing the re-establishment of a functional CarG/SRF/myocardin complex and the expression of differentiation genes [103].

In spontaneously hypertensive (SHR) rats, aerobic exercise suppresses the vSMCs’ phenotypic switch via Akt and mitogen-activated protein kinase (MAPK) signalling. Aerobic exercise decreases blood pressure and reverses the decreased expression of the vSMC protein markers (calponin, SM α-actin and osteopontin) induced by hypertension. Thus, hypertension, sedentary habits and a lack of exercise in the general population might promote atherogenesis due to an inhibition of Akt/MAPK signalling pathways. Akt inhibition in fact significantly inhibits the expression of contractile proteins, which is instead increased by p38 MAPK and ERK inhibition. The latter in addition downregulates the expression of the synthetic phenotype marker, osteopontin [104]. The effects of aerobic exercise suggest the implication of redox signalling in the maintenance of the vSMC contractile phenotype as well as in the hyperplastic response associated with vascular injury. Kinases are downstream targets of ROS produced by enzymes, such as the NOX complex, and NOX-4 was in fact shown to contribute to the activation of MAPK-14 and the suppression of the vSMCs contractile phenotype [105].

The important role of PI3K/Akt signalling in survival, proliferation, polarisation and migration of macrophages has been previously reviewed [106]. The activity of Akt signalling determines monocyte/macrophage viability and their resistance to pro-apoptotic stimuli in atherosclerotic lesions. A selective inhibition of Akt and mTOR was indeed shown to increase plaque stability through the promotion of macrophage autophagy in a rabbit model of atherosclerosis [107].

The PI3K/Akt/mTOR pathway is implicated in vSMC autophagy as well. In ApoE^−/−^ mice, the inhibition of P2Y12 purinergic receptor promotes vSMC autophagy through the PI3K/Akt/mTOR pathway. Conversely, the P2RY12 receptor activation can block cholesterol efflux and macrophage autophagy, thus promoting the formation of vSMC-derived foam cells [108]. The activation of the PI3K/Akt/mTOR pathway induced by homocysteinaemia was also prevented by miR-145, thus inhibiting vSMC proliferation, migration and phenotypic switch [109].

NO signalling is intimately linked to the PI3K/Akt/mTOR pathway. Liu et al. demonstrated that a NO donor, PABA/NO (O^2^-{2,4-dinitro-5-[4-(N-methylamino) benzoyloxy] phenyl}1-(N,N-dimethylamino)diazen-1-ium-1,2-diolate) induces apoptosis in hepatocarcinoma cells through the inhibition of the PI3K/Akt/mTOR and MEK/ERK pathways. Data suggest that PABA/NO could be used as a potential therapeutic through the regulation of this pathway also in other pathologies, such as atherosclerosis [110]. Moreover, it was shown both in vivo and in vitro that the specific inhibition of PI3K effected by high levels of luteinizing hormone (LH) results in the suppression of eNOS-dependent production of NO, which is accompanied by promotion of atherosclerosis progression [111].

### 3.2. Raf/Ras/MEK/ERK Signalling

The Raf/Ras/MEK/ERK pathway—driven by the MAPK cascade as well as by ubiquitous growth factors such as PDGF-BB, epidermal growth factor (EGF) or fibroblast growth factor (FGF)—is considered as the master inducer of de-differentiation. It in fact promotes Elk-1 phosphorylation, leading to the expression of genes implicated in vSMC growth and proliferation and to the repression of genes coding for vSMC markers [112]. The Raf/Ras/MEK/ERK pathway has an important role in the maintenance of lipid balance and foam cells formation. Hu et al. investigated the activity, abundance and localisation of ERK1/2 in atherosclerotic lesions of cholesterol-fed rabbits [113]. Immunofluorescent analysis revealed an abundant and heterogeneous distribution of ERK1/2 in the cap and basal regions of atheromas. ERK1/2 was heavily phosphorylated on tyrosyl residues and co-expressed with proliferating cell nuclear antigen in atherosclerotic lesions. Furthermore, vSMCs derived from atherosclerotic lesions showed increased migratory/proliferative ability and higher ERK activity in response to LDL stimulation, compared to cells extracted from non-atherosclerotic vessels. These results suggest that a persistent activation and overexpression of ERK1/2 could be an initiator of cell proliferation during atherosclerosis progression and perpetuate the process in time [113]. The inhibition of ERK1/2 is a promising treatment because it reduces lipid deposition, upregulates the expression of ABCA1/G1 lipid efflux transporters and suppresses the expression of CD36 in oxLDL‑stimulated macrophages [114].

## 4. Transcriptional Regulation of Phenotypes

### 4.1. Activator Protein-1 (AP-1)

AP-1 is the main physiological inhibitor of tissue-type plasminogen activator (t-PA), and therefore an important inhibitor of fibrinolysis. AP-1 controls several cellular events including cell proliferation, differentiation and apoptosis [115]. Previous studies showed that AP-1 activity is negatively affected by *S*-nitrosation and increased by antioxidants. AP-1 activity is in fact related with thioredoxin levels, suggesting a redox regulation of AP-1 [116]. Another study showed that the downregulation of NF-κB and AP-1 in vSMCs allows the inhibition of MMP-9 expression and induces G1-cell cycle arrest via PTEN [117]. PTEN is an inhibitor of the PI3K/Akt pathway and an inositol phosphatase, known to inhibit PDGF-mediated vSMC proliferation and migration [118]. Overexpression of PTEN inhibits growth factor-induced activation of Akt and p70 and proliferation, migration and survival of vSMCs. Furthermore, a critical role of AP-1 was shown in the atherosclerosis-associated process termed called arteriogenesis. The latter consists of wall remodelling and proliferation of collateral arterioles and is promoted by monocytes recruited from blood by the monocyte chemoattractant protein-1 (MCP-1). AP-1 in fact mediates an increased MCP-1 expression in vSMCs under stretch stress [119].

### 4.2. Nuclear Factor Erythroid 2-Related Factor (Nrf-2)

Nrf-2 is a transcription factor, member of the cap’n’collar (CNC) subfamily, mediating the induction by antioxidants and electrophiles of detoxifying enzymes such as glutathione *S*-transferases or NAD(P)H:quinone oxidoreductase-1 (NQO-1) [120,121].

During atherogenesis, Nrf-2 can play a twofold role. On the one hand, it is pro-atherogenic, since Nrf-2 is the second transcription factor—after PPAR-γ—involved in oxLDL-induced expression of CD36 and antioxidant stress proteins (A170, heme oxygenase-1, peroxiredoxin I) in macrophages [122]. Furthermore, Nrf-2 is a key regulator of Mox macrophages developed in response to oxidative tissue damage [4]. Treatment of M1 and M2 macrophages with oxidized phospholipids in fact induces their phenotypic switch to Mox macrophages through a marked Nrf-2-mediated expression of redox-regulatory genes.

On the other hand, there are anti-atherosclerotic mechanisms. Both Nrf-2 and Keap-1 are key components of the oxidative stress response for the maintenance of vascular homeostasis, through the induction of vSMC apoptosis and inhibition of neointimal hyperplasia [123]. Nrf-2 also participates in anti-atherosclerotic adaptive defence mechanisms, by inducing heme oxygenase-1 (HO-1) expression [124]. Monocyte-derived macrophages of healthy subjects in fact exhibit a lower oxidative stress status as compared to coronary artery disease patients, along with lower levels of Nrf-2 and HO-1. The latter are instead highly expressed in macrophages of active plaques. Thus, the HO-1 levels may reflect plaque vulnerability and allow the identification of patients with rupture-prone plaques [124].

As far as NO connections are concerned, earlier studies documented that NO itself is a potent inducer of HO-1 in vSMCs [125,126], and the effect was indeed shown to be mediated through activation of the Nrf-2/ARE axis [127]. It was suggested that the phenomenon may represent a critical adaptive response to maintain cell viability at sites of vascular inflammation during atherosclerosis.

### 4.3. Nuclear Factor-κB (NF-κB)

NF-κB is a key regulator of the inflammatory response. It is usually sequestered in the cytoplasm in complexes with IkBs, a family of inhibitory proteins. NF-κB translocation to the nucleus is thus prevented, avoiding the expression of genes encoding inflammatory and immuno-modulatory proteins, as well as of genes regulating cell differentiation, survival and proliferation [128]. The NF-κB family, encoded by the Rel gene family, is composed of five members—p50, p52, p65 (RelA), c-Rel and RelB—as well as six IkB members, which emphasizes its complex regulation. c-Rel allows macrophages to scale the inflammatory response after a transient or persistent stimulation of toll like receptor 4 (TLR-4), which also depends on macrophages localisation in tissue vs. blood [129,130]. p65 signalling was shown to directly upregulate the promoter activity of miR-17, an inducer of cell cycle G1/S transition and vSMCs proliferation. As NF-κB is both activated by inflammation and regulates inflammation, p65-dependent miR-17 upregulation could represent a mechanism explaining the excessive proliferation of vSMCs [131].

Chylomicron remnants and oxLDLs, both inducing foam cells formation, have been implicated in the suppression of NF-κB activity in macrophages [132]. Indeed, oxLDLs mediate oxidative modifications of NF-κB, such as carbonylation of the p65 subunit [133]. The uptake of oxLDLs is highly reduced in activated p50-deficient macrophages, along with a prolonged production of TNF-α in response to lipopolysaccharides (LPS) [134].

**Figure 2:**
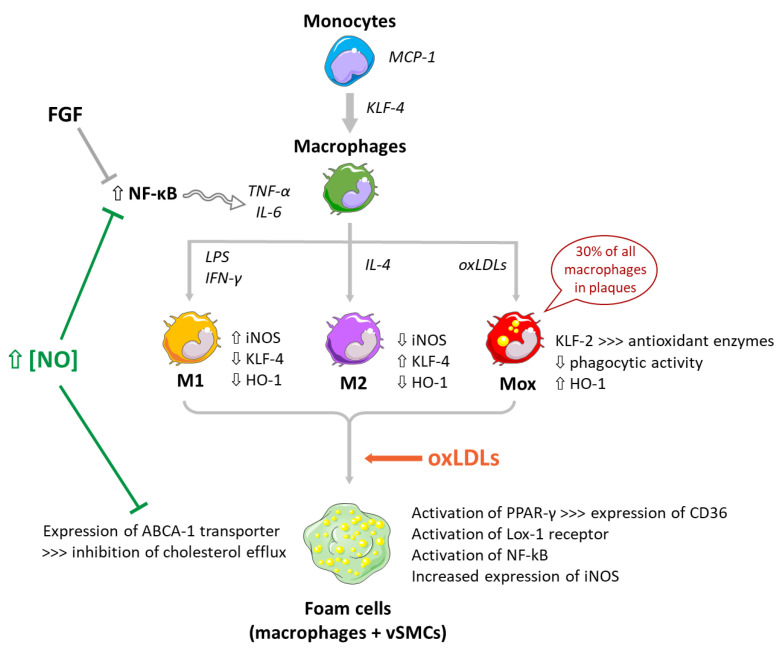
Involvement of NO and oxLDLs in the differentiation of monocytes to different subtypes of macrophages and formation of foam cells. NO: nitric oxide; iNOS: inducible NO synthase; FGF: fibroblast growth factor; NF-κB: nuclear factor-κB; TNF-α: tumour necrosis factor alpha; IL-6: interleukin-6; TLR4: toll-like receptor 4; MCP-1: monocyte chemoattractant protein 1; KLF-4: Krüppel-like factor 4; LPS: lipopolysaccharide, IFN-γ: interferon-γ; IL-4: interleukin-4; HO-1: heme oxygenase 1; KLF-2: Krüppel-like factor 2; LDLs: low-density lipoproteins; oxLDLs: oxidized low-density lipoproteins; PPAR-γ: proliferator-activated receptor γ.

The effects of NO on NF-κB functions are biphasic. In the first place, high NO concentrations can be pro-apoptotic and pro-atherosclerotic, by inhibiting NF-κB activation in macrophages, monocytes and neutrophils. NF-κB DNA-binding is in fact inhibited by *S*-nitrosation of the p50 subunit (Figure 2). NO also inhibits NF-κB activation in rat vSMCs via a cGMP-independent inhibition of IκB phosphorylation. NF-κB activity is also stimulated through the nitration of IκB.strα following activation of NO synthase [135]. On the other hand, NO can also have anti-apoptotic effects, through modulation of several proteins of the Bcl-2 family via sGC activation or inhibition by *S*-nitrosation of a large number of apoptotic proteins (e.g., caspases) [136].

### 4.4. Myocardin/Serum Response Factor (SRF)

**Figure 3:**
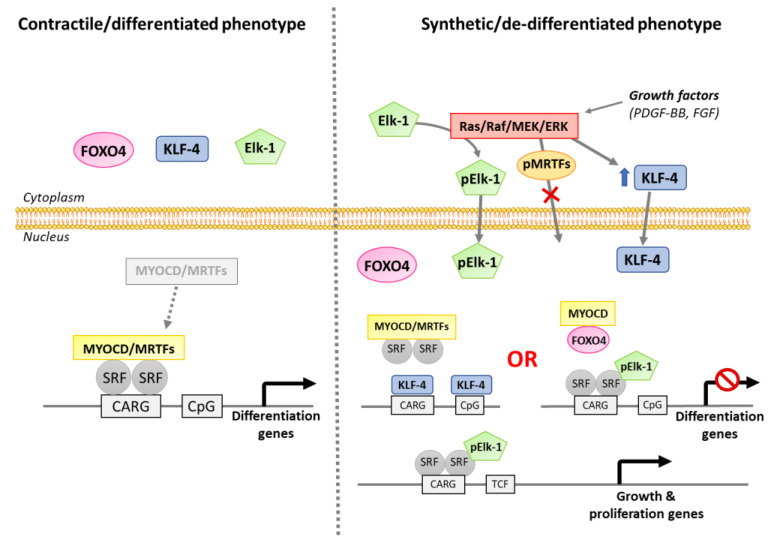
Impact of the myocardin–SRF–CarG complex and its cofactors in the maintenance vs. de-differentiation of the vSMCs’ phenotype. CARG: CarG-box sequence; MYOCD: myocardin; MRTFs: myocardin-related transcription factors; SRF: serum response factor; KLF-4: Krüppel-like factor 4; PDGF-BB: platelet-derived growth factor-BB; FGF: fibroblast growth factor; TCF: ternary complex factor.

Myocardin is a powerful myogenic transcriptional regulator and co-activator, specifically expressed during adulthood in smooth muscle and myocardium. It negatively regulates vSMC inflammatory activation and reduces monocytes infiltration [137]. Myocardin is closely associated with SRF, considered as a docking platform for its activity. SRF is a ubiquitous DNA-binding transcription factor binding to a 10-base-pair CarG-box sequence (CC(A/T-rich)_6_CC), and activate the transcription of genes implicated in muscle differentiation and proliferation [138,139]. The myocardin/SRF/CarG complex is disrupted during the vSMC de-differentiation process. KLF-4, Elk-1 or FOXO-4 transcription factors act as co-repressor proteins, which compete with the myocardin/SRF/CarG complex and prevent the expression of differentiation genes [103] (Figure 3). Phosphorylated Elk-1 displaces myocardin from its SRF docking site and replaces it to form a nuclear factor activating the transcription of growth and proliferation genes.

Myocardin levels are associated with the vSMCs’ phenotypic switch and are decreased during progression of atherosclerosis. In ApoE^−^/^−^ mice, myocardin deficiency was shown to accelerate atherogenesis. On the contrary, increased myocardin levels can prevent the expression of inflammatory cytokines, chemokines and adhesion molecules in vSMCs [137]. Thus, endogenous levels of myocardin are an important regulator of vessel inflammation, which identifies this transcriptional regulator as “the guardian” of the vSMCs’ contractile phenotype.

NO can participate in the modulation of myocardin functions, with important bearings on vSMCs phenotypic switch and neo-angiogenesis. Vascular endothelial growth factor A (VEGF-A) was in fact shown to promote myocardin *S*-nitrosation, resulting in STAT-3 activation and vSMC proliferation, along with the inhibition of the vSMC markers’ expression, such as ACTA-2, SM-22 and Myh-11. In addition, in experiments using GSNO as a NO donor, it was shown that myocardin can dose-dependently enhance the expression of GSNO reductase (GSNOR, also known as alcohol dehydrogenase class III), the enzyme effecting the catabolism GSNO into GSNHOH, thus downregulating NO bioavailability within the cell [140].

### 4.5. Krüppel-Like Factor 4 (KLF-4)

KLF-4 is a pluripotent transcription factor, physiologically absent from contractile vSMCs but rapidly upregulated in response to vascular injury [141]. KLF-4 is a transcriptional target of BMPs 2, 4 and 6 as well as TGF-β1, resulting in the modulation of vSMC differentiation. Under vascular injury, KLF-4 is upregulated and mediates the vSMCs’ phenotypic switch in response to TGF-β1 through the activation of Smad2/3 and p38 MAPK signalling. KLF-4-promoted vSMC de-differentiation proceeds through the inhibition of SM-α-actin, SM22α and SM-MHC expression, inducing vascular remodelling and plaque progression [142,143]. Furthermore, KLF-4 represses vSMC genes through the downregulation of myocardin and prevents the association of the SRF/myocardin complex with gene promoters of phenotypic vSMC markers (SM22α, SM-MHC). Vendrov et al. demonstrated in mice that KLF-4 activates pro-inflammatory genes and allows vSMCs to acquire a pro-inflammatory, macrophage-like phenotype [144]. They also studied the effect of NOX activator A (NOXA1) deletion on the vSMCs’ phenotype. NOXA1 is a functional homolog of p67-phox in activation of NADPH oxidase, and its deletion therefore decreases vascular levels of ROS and the size of the atherosclerotic lesions. The SMC-specific deletion of NOXA1 in mice produced a marked decrease in ROS generation, in TNF-α-induced vSMCs proliferation and migration, as well as in CD68^+^ cells, myosin expression and levels of KLF-4 with its downstream targets (VCAM, MMP2). These results support an important role of NOXA1-dependent NAPDH oxidase activity in vSMC plasticity, acting by increasing vSMC proliferation and migration, KLF-4-mediated transition to a macrophage-like phenotype and plaque inflammation. The stimulating protein-1 (Sp-1) transcription factor was shown to be essential for KLF-4 activation by PDGF-BB [145]. Indeed, Sp-1 overexpression can itself increase KLF-4 promotor activity and vSMCs phenotype modulation. In addition, following activation by oxidized phospholipids, KLF-4 mediates the upregulation of several extracellular matrix genes, such as type VIII collagen, and promotes vSMCs migration, contributing to progression of atherosclerosis [146].

During the differentiation of monocytes into macrophages, KLF-4 is regulated through demethylation of its promoter by a cytidine deaminase [147]. KLF-4 upregulates ApoE, an important anti-atherosclerotic factor [148], and is a critical regulator of macrophage polarisation [149], being highly expressed in M2 macrophages and downregulated in M1 macrophages (Figure 2). It has been recently shown in endothelial cells that KLF-4 can undergo *S*-nitrosation in response to a nitrosative stress, which impairs its activity [150]. Indeed, after a treatment with the physiological NO-donor *S*-nitrosoglutathione (GSNO), the KLF-4-dependent vasodilatory response in pulmonary arteries is impaired.

## 5. Conclusions

**Figure 4:**
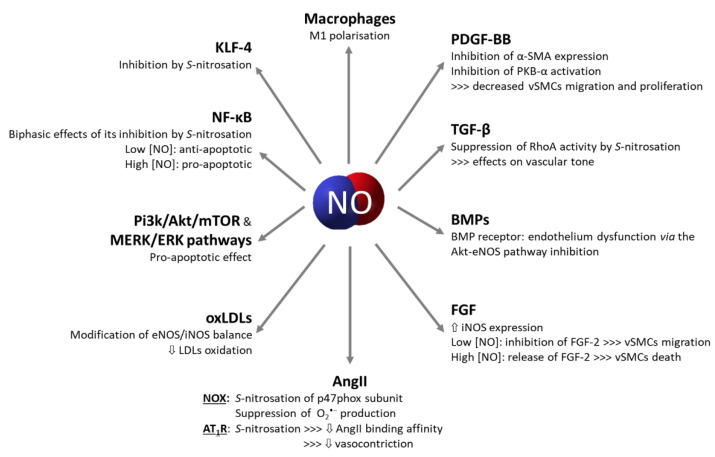
Mechanisms and pathways impacted by NO bioavailability. NO: nitric oxide; PDGF-BB: platelet-derived growth factor-BB; vSMCs: vascular smooth muscle cells; TGF-β: transforming growth factor-β; iNOS: inducible NO synthase; BMPs: bone morphogenic proteins; eNOS: endothelial NO synthase; FGF: fibroblast growth factor; AngII: angiotensin II; AT_1_R: angiotensin II type I receptor; NOX: NAPDH oxidase; O_2_^•−^ superoxide anion; oxLDLs: oxidized low-density lipoproteins; LDLs: low-density lipoproteins; NF-κB: nuclear factor-κB; KLF-4: Krüppel-like factor 4.

vSMCs and macrophages are the main cells implicated in atherosclerosis development, and their phenotypes are modified/switched upon various stimuli. Differentiation or de-differentiation processes implicate a large number of mechanisms and signalling pathways. In the last decade, several studies attempted to better understand these processes and to explore their possible utilisation in therapeutics. From this perspective, studies have highlighted the importance of NO in the progression of atherosclerosis, with a biphasic effect on cells depending on its concentration. NO can act through *S*-nitrosation to modulate the pathways discussed above (Figure 4). The differentiation of monocytes/macrophages and their phenotypic switch are sufficiently well characterised, while the signalling pathways driving the de-differentiation of the vSMCs remain partly unclear. While the implication of NO in atherosclerosis progression is indubitable, the interconnections between NO signalling and other relevant signalling pathways remain poorly understood. The identification of these interconnections could help to identify novel specific therapeutic targets, to be possibly exploited in synergy with other current anti-atherosclerotic strategies (hypolipidemic agents or antiplatelet drugs).
